# Increased yield stability of field-grown winter barley (*Hordeum vulgare* L.) varietal mixtures through ecological processes

**DOI:** 10.1016/j.cropro.2016.03.001

**Published:** 2016-07

**Authors:** Henry E. Creissen, Tove H. Jorgensen, James K.M. Brown

**Affiliations:** a Crop Genetics Department, John Innes Centre, Norwich Research Park, Norwich, Norfolk, NR4 7UH, UK; b School of Biological Sciences, University of East Anglia, Norwich, Norfolk, NR4 7TJ, UK; c Department of Bioscience, Aarhus University, 8000 Aarhus C, Denmark

**Keywords:** *Puccinia hordei*, Crop disease, Compensation, Facilitation, Variety mixture, Barley

## Abstract

Crop variety mixtures have the potential to increase yield stability in highly variable and unpredictable environments, yet knowledge of the specific mechanisms underlying enhanced yield stability has been limited. Ecological processes in genetically diverse crops were investigated by conducting field trials with winter barley varieties (*Hordeum vulgare*), grown as monocultures or as three-way mixtures in fungicide treated and untreated plots at three sites. Mixtures achieved yields comparable to the best performing monocultures whilst enhancing yield stability despite being subject to multiple predicted and unpredicted abiotic and biotic stresses including brown rust (*Puccinia hordei*) and lodging. There was compensation through competitive release because the most competitive variety overyielded in mixtures thereby compensating for less competitive varieties. Facilitation was also identified as an important ecological process within mixtures by reducing lodging. This study indicates that crop varietal mixtures have the capacity to stabilise productivity even when environmental conditions and stresses are not predicted in advance. Varietal mixtures provide a means of increasing crop genetic diversity without the need for extensive breeding efforts. They may confer enhanced resilience to environmental stresses and thus be a desirable component of future cropping systems for sustainable arable farming.

## Highlights

• Variety mixtures were less diseased and less prone to lodging than monocultures.
• Mixtures were more tolerant of unexpected environmental stresses.
• Yields were more stable in mixtures than monocultures.
• Mixture success was due to ecological processes: compensation and facilitation.
• It is possible to fine-tune mixtures to suit specific growing conditions.

## 1 Introduction

An alternative to the variety monoculture system is the use of varietal mixtures in which several genotypes are sown together in the same field to buffer against environmental stresses, including disease, and to increase yield stability (Wolfe, 1985, Lannou and Mundt, 1996, Zhu et al., 2000). To date, varietal mixtures have primarily been deployed against crop diseases, controlling major pathogens such as powdery mildew of barley (Wolfe and Barrett, 1980), Rhynchosporium scald of barley (Newton et al., 1997), wheat yellow rust (Sapoukhina et al., 2013), and rice blast (Zhu et al., 2000). Mixtures can reduce disease severity by reducing pathogen spread, either by increasing the distance between susceptible host plants, or by resistant plants forming a barrier to prevent pathogen dispersal (Chin and Wolfe, 1984, Zhu et al., 2000).

The theory underpinning the use of mixtures is largely based on the hypothesis that biodiversity increases ecological stability (Yachi and Loreau, 1999). This approach relies on beneficial ecological processes to increase the system's potential to buffer against adverse environmental conditions, reduce fertiliser inputs and control disease (Finckh and Wolfe, 1998). Variation between mixture components in response to common pathogens allows ecologically beneficial processes such as compensation, complementation and facilitation to occur (Wolfe, 1985). Complementation between crop plants can increase productivity in mixtures through niche differentiation and resource partitioning (Loreau, 2000, Mulder et al., 2001, Tilman, 2004). Facilitation can occur within mixed populations if the fitness of neighbouring plants is increased through inter-plant interactions such as provision of shade and deterrence of pests (Callaway, 1995). When weaker individuals are harmed by environmental stress, stronger plants can increase their yields through compensation via competitive release (a reduction in competition) (Tilman, 1996, Creissen et al., 2013, 2015).

Compensation is thought to be the major ecological process contributing to yield stability in diverse mixtures (Eberhart and Russell, 1966, Wolfe, 1985, Smithson and Lenne, 1996, Mundt, 2002, Østergård et al., 2005, Cowger and Weisz, 2008) but other beneficial processes may also be involved (Finckh et al., 2000). For example, competitive release allowed a mixture of high yielding wheat varieties and winter hardy varieties to insure against excessive losses in colder winters, because stress-tolerant plants overyielded in those conditions (Finckh et al., 2000). The potential to exploit ecological processes that generate beneficial plant–plant interactions therefore depends on the presence of suitable varieties as mixture components. Field trials are necessary for accurate mixture assessment as it is often difficult to predict the performance of a variety in mixture from its monoculture yield due to the complexity of ecological interactions taking place within the crop and the variability of field environments (Lopez and Mundt, 2000, Mille et al., 2006).

When varietal mixture studies are conducted in only one type of environment, as is often the case, the strength of any conclusions is limited because they cannot necessarily be extrapolated to other environments (Mundt, 2002). In contrast, replicated trials across multiple sites reveal consistencies across environments as well as the environmental dependency of interactions within the mixture. The scale of environmental variation determines the scope of the relevance of results of such trials. Some replicated trials have studied variety mixture performance across highly dissimilar climates (Østergård et al., 2005) or across a range of disease pressures including artificially high infection (Finckh et al., 2000). In order to optimise variety mixtures for actual farming situations, by contrast, it is necessary to understand how their performance is affected by relatively fine-scale environmental variation between fields or by apparently minor variation in the composition of the mixture.

This study examines the ecological processes that stabilise the yields of varietal mixtures in replicated populations under natural, fine scale levels of environmental stress and disease. We test the hypotheses that compensation by better-adapted plants increases yield stability in phenotypically diverse mixtures, and that this effect is greatest when susceptible and resistant varieties are combined under natural levels of infection by pathogens. We test the prediction that GxE interactions will alter the competitive ability and fitness of individual varieties, yet the overall mixture yield will be maintained through stabilising ecological processes such as compensation, complementation and facilitation.

## 2 Materials and methods

### 2.1 Mixture design

UK commercial varieties of winter barley (*Hordeum vulgare*) were selected for our experiments based on phenotypic information contained in the HGCA (Home Grown Cereals Authority, now part of the Agriculture and Horticulture Development Board) Recommended List for 2011/2012 (http://cereals.ahdb.org.uk/media/4287/wbrl-11.pdf). Two mixtures were designed to contain three varieties varying in disease resistance, competitive ability and classification group which is based on morphological differences in the ear (two-row or six-row) and the crop's end-usage (malting or feed). Each variety had a set of unique phenotypic traits for easy identification in the field. Mixtures contained the hybrid six-row Element, the red-awned two-row Winsome, and a white grain two-row variety which was either Cassata or Saffron. Variation in mixture composition was restricted so that the effect of relatively small changes in mixture composition on ecological processes and agronomic performance could be assessed.

Information from previous trials at the John Innes Centre (JIC) and observations from commercial farms in Norfolk suggested that the fungal diseases Rhynchosporium scald (caused by *Rhynchosporium commune*) and brown rust (*Puccinia hordei*) were the major biotic threats to crop yield. For the present study, each mixture therefore contained one variety susceptible to common genotypes of either disease based on HGCA resistance ratings (Table 1). Saffron was predicted to be the most susceptible variety to Rhynchosporium (Mixture A). Element was present in both mixtures but was the most susceptible variety to brown rust in Mixture B (Table 1) because other varieties in the mixture possessed good resistance to both brown rust and Rhynchosporium.

Target plant populations were set according to plant breeding companies' recommendations of 300 plants/m^2^ for the two-row varieties, and 200 plants/m^2^ for the six-row variety. For each mixture plot, one third of the seed required for each variety's monoculture plot (100 plants/m^2^ for the 2-rows, 67 plants/m^2^ for the six-row) was thoroughly mixed by hand, prior to sowing in the field with a Hege 80 drill in an six row format (Wintersteiger, Austria). All plots were 6 m^2^ (1.5 m × 4 m).

### 2.2 Trial sites and experimental design

Trials were sown on 30^th^ September 2011 at three sites with different soil types and representing different edaphic conditions in Norfolk, UK. Sites were located within three miles of one another and so experienced similar climatic conditions. Soil types ranged from a very light sandy clay loam (JIC; GPS 52.6225, 1.2184), to a light sandy clay loam (‘light’: Bawburgh; GPS 52.6251, 1.1745), and a heavy sandy clay loam (‘heavy’: Bawburgh; GPS 52.6287, 1.17862). The preceding crop for both the ‘light’ and ‘heavy’ site was winter beans (soil index of N2, P3, K1, pH 7.5), whereas at the JIC site the land was fallow (soil index N1, P3, K1, pH 7.6). All plots received Nitrogen 40 kg/ha and Sulphur 45 kg/ha on 2nd March 2012, and Nitrogen 100 kg/ha, Phosphorous 54 kg/ha and Potassium 108 kg/ha on 30th March 2012. The experiments at each site consisted of four replicates of each monoculture and each mixture (A and B) with two treatments (fungicide and no fungicide) in a randomised complete block design, giving 48 plots per site and 144 plots in total.

### 2.3 Chemical treatments

Chemical treatments were based on recommendations from a local agronomist and applied manually using a knapsack sprayer at the manufacturers’ recommended rates. All plots received the herbicides Ally Max and Oxytril at T1 (Growth Stage, GS 30–32; Zadoks et al., 1974) and the plant growth regulator Chlormequat at T2 (GS 32–37). Non-disease control plots received a full fungicide treatment programme consisting of full rate applications of Opus (epoxiconazole) at T1 and T2, and Proline (prothioconazole) at T3 (GS39-45) as broad-spectrum fungicides, Bravo (chlorothalonil) to control Rhynchosporium at T1 and T2, Cyflamid (cyflufenamid) against mildew at T1, and Comet 200 (pyraclostrobin) against rusts at T2 and T3.

### 2.4 Disease scoring

Disease levels were visually assessed at GS49-50 when symptoms were at their maximum. Disease was measured as % green leaf area covered in symptoms on the flag and second leaf (Peterson et al., 1948, James et al., 1968, James, 1971). Ten plants of each variety were scored per plot. Plants on the outer rows were not scored to avoid edge effects. Diseases scored included brown rust, powdery mildew (*Blumeria graminis* f. sp. *hordei*), Rhynchosporium and net blotch (*Pyrenophora teres*).

### 2.5 Plant height measurement

Maximum plant height measurements were taken at GS 30–32 when height differences were greatest between sites and plots. Height measurements were taken from ten randomly selected individual plants from within each plot, as it was impossible to identify individual varieties within mixture plots at this early stage. Plants on the outer rows were not measured to avoid edge effects. Lodging was scored by eye as % of plants in a plot that were fully lodged (75–90° from upright), rounded to the nearest one-eighth of a plot, ten days prior to harvest.

### 2.6 Yield measurements

Total plot yield (g) (adjusted to 15% grain humidity) was recorded at harvest and yield component measurements including seed mass/ear, number of seeds/ear and average seed mass/ear were taken. For each mixture plot 100 grains were randomly selected for varietal identification through analysis of visually assessable phenotypic characteristics by the National Institute of Agricultural Botany (NIAB, Table A1). These data were used to estimate the relative proportions (%) contributed by each variety to the total mixture yield (g). Mean relative yields (RY = yield in mixture/monoculture) (de Wit, 1960) were calculated for each variety at each site under both fungicide and non-fungicide treatments as follows: the estimated proportions contributed by each variety to the overall mixture plot yield (%) were multiplied by the total mixture plot yield (g), and this value was then divided by the average plot yield of that variety in monoculture.

### 2.7 Yield stability assessments

Yield stability was estimated by Wricke's (1962) ecovalence, *W*^*2*^ which is a measure of dynamic rather than static stability (Annicchiarico, 2002). Whereas static stability statistics describe a constant yield across environments, even if the yield is low, *W*^*2*^ measures the GxE interaction effect for a particular genotype and is therefore commonly used to assess if a set of genotypes maintains the same relative contributions to total yield in different environments (Becker and Léon, 1988, Annicchiarico, 2002). In this study, *W* allows the effect of the environment on yield stability to be compared in different barley varieties and mixtures. *W*^*2*^ was calculated for each genotype in each treatment (monocultures and mixtures) separately and then averaged across genotypes in the mixtures to compare yield stability between treatments. The contribution (*W*_*ij*_^*2*^) of genotype *i* in environment *j* to *W*^*2*^ was calculated as:

$$W_{ij}^{2}{=\text{Σ}}_{i,j}{\left(X_{ij}-{\bar{X}}_{i.}-{\bar{X}}_{.j}+{\bar{X}}_{..}\right)}^{2}$$

where *X*_*ij*_ is the yield of *i* in *j*, ${\bar{X}}_{i.}$ is yield of genotype *i* averaged across environments, ${\bar{X}}_{.j}$ is yield averaged across genotypes in environment *j* and ${\bar{X}}_{..}$ is the grand mean. *W*^*2*^ = 0 (the lowest possible value) indicates high yield stability and an absence of GxE interaction.

### 2.8 Statistical analysis

Linear mixed modelling was used to evaluate differences in yield and disease between monocultures and mixtures of winter barley varieties. Plot yield, disease scores, ear mass, mean seed mass/ear and mean number of seed/ear were analysed in separate models all including the main effects of variety, site and cultivation (monoculture/mixture) as fixed factors and all interactions between them. The plot in which the plants were grown was included as a random effect. All non-significant (*P* > 0.05, *F*-test) interactions between the main terms were removed from the analysis. Statistical analysis was conducted using Genstat v.14 (VSN International, 2011).

## 3 Results

### 3.1 Yield

**Fig. 1:**
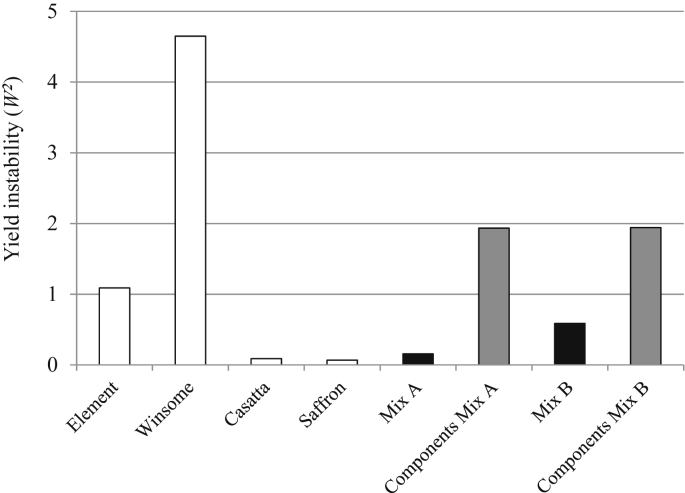
Yield stability reported using Wricke's ecovalence (*W*^*2*^) for 6 m^2^ plot yields of winter barley monocultures and mixtures in field trials at three environmentally diverse sites. A score of 0 indicates the greatest possible level of stability. White bars estimate stability in monocultures (N = 24/monoculture). Black bar estimate stability in mixtures (N = 24/mixture). Each grey bar reports the means of 3 stability estimates for varieties grown in monoculture that formed components of the mixture. N = 144 plots in total (N = 24 per monoculture and mixture).

**Fig. 2:**
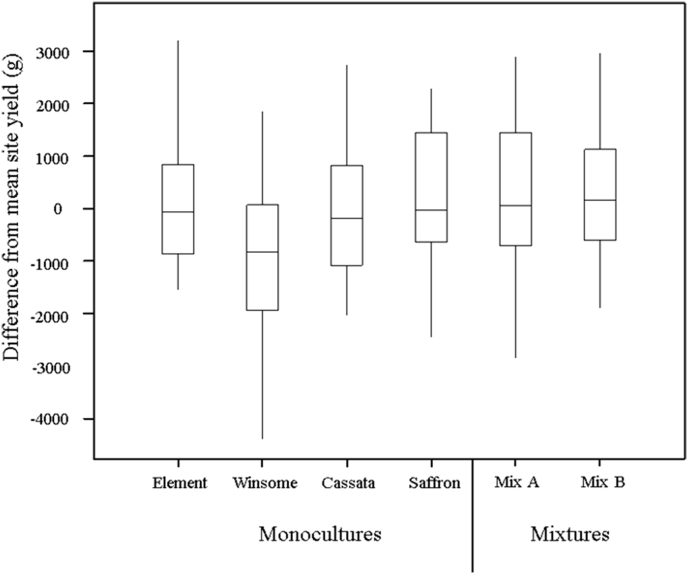
Difference from mean site yield (g) for each monoculture and mixture in a winter barley field trial experiment conducted over three different sites. Mixture A includes varieties Element, Winsome and Saffron. Mixture B includes varieties Element, Winsome and Cassata. The bottom and top of the boxes represent the first and third quartiles. Lines within the box represent the median. Lines outside the box display the range. N = 144 (N = 24 per monoculture/mixture).

**Fig. 3:**
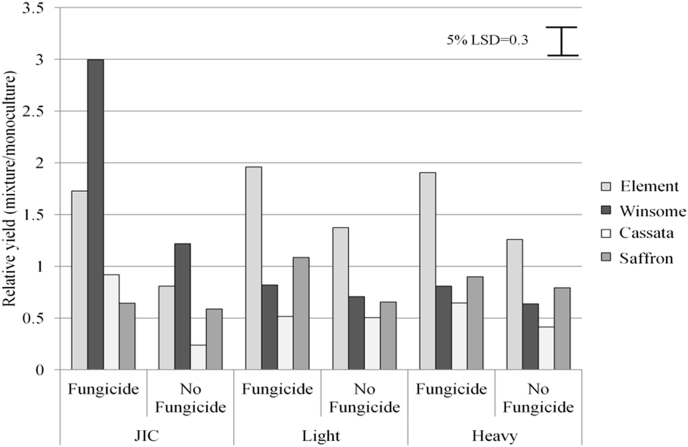
Relative yields of four winter barley varieties grown in mixtures in fungicide treated or untreated plots in a field trial conducted over three different sites. Relative yield was calculated by multiplying proportions contributed by each variety to the overall mixture plot yield by the total mixture plot yield (g), and dividing by the average plot yield of that variety in monoculture). Error bar shows Least Significant Difference at the 5% level. N = 24 per monoculture.

Mean yields of mixtures and monocultures were similar across the entire experiment (Table A2, F_3,10_ = 2.23, *P* = 0.1) but mixture yields were more stable than monoculture yields as shown by lower values of *W*^*2*^ in the mixtures compared to the mean of their component varieties grown as monocultures (Fig. 1). Mixture yields were stable, despite the presence of a variety with highly variable yields, Winsome, in both mixtures (Fig. 1, Fig. 2). Mixture performance of each variety was altered by the site and fungicide treatment, shown by changes in relative yield (Fig. 3). Mixture B was more stable than any monoculture but less stable than Mixture A (Fig. 1) indicating that relatively minor modifications to mixture composition by the substitution of a single variety in a three-way mixture can change the outcome of the ecological processes and the resulting agronomic performance of the mixture. Element generally performed better in mixture indicating that inter-plant competition was greater within monocultures (Fig. 3). The success of Element in mixtures appeared to be partly due to plasticity in certain yield components, because the variety tended to have greater mean ear mass in mixture (Table A3a, F_3,9_ = 3.6, *P* = 0.01). Plasticity in mean ear mass data was dependent on cultivation (mixture/monoculture) but not site (Table A3a, F_1,9_ = 0.42, *P* = 0.5). Cassata's low competitive ability in mixtures led to a reduction in yield and yield components including mean mass per ear (Fig. 3, Table A3a, F_3,9_ = 3.6, *P* = 0.01). Element overyielded in mixture and thus compensated for under-yielding varieties, resulting in high and stable mixture yields across the entire experiment (Fig. 2, Fig. 3).

### 3.2 Disease

The disease levels observed in our study matched disease resistance ratings on the HGCA recommended list 2011–12 with one exception; Winsome (brown rust rating = 6) had significantly more brown rust than Element (brown rust rating = 4) at JIC and in the light land trial. Brown rust was by far the most prevalent disease, assessed by total green area covered in disease on the flag and second leaves at the end of the growing season. Powdery mildew and net blotch infections were recorded but were at such low levels that it was not possible to analyse them statistically. Unexpectedly, the least prevalent disease was Rhynchosporium scald with only six plants showing signs of infection (data not shown).

**Fig. 4:**
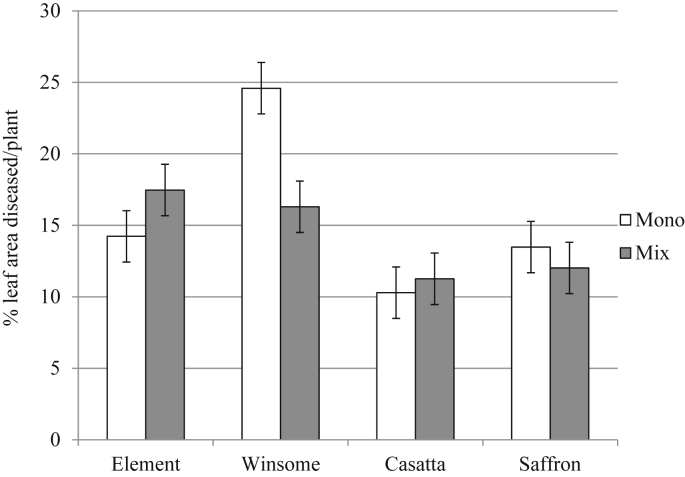
Mean green leaf area (%) on the flag and second leaves diseased by brown rust. Disease scores are for individual plants grown in monoculture or three-way mixture and naturally infected under field conditions. Specific data for individual sites is not shown because there was no significant interaction between site and cultivation (mixture/monoculture). N = 1680. Error bars show 95% confidence interval of means.

Brown rust scores, recorded as the % leaf area infected with the fungus, were heavily dependent upon interactions between variety and cultivation (mixture/monoculture) (Fig. 4; Table A4, F_3,15_ = 4.31, *P* = 0.006). Disease levels were consistently reduced for Winsome when grown in mixture compared to monoculture, indicating a positive effect of mixtures in reducing disease severity for susceptible varieties. There was no significant difference in disease levels between the different mixtures because the most susceptible varieties, Winsome and Element, were present in both mixtures (data not shown).

**Fig. 5:**
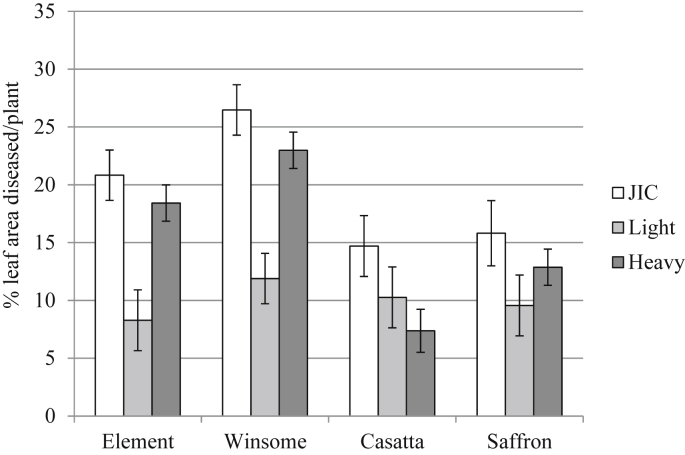
Mean % green leaf area on the flag and second leaves diseased by brown rust. Disease scores for individual plants grown in mixture and monoculture and naturally infected under field conditions conducted at three sites located in Norfolk. JIC = John Innes Centre site. Light = light land trial site at Bawburgh. Heavy = heavy land trial site at Bawburgh. N = 1680. Error bars show 95% confidence interval of means.

**Fig. 6:**
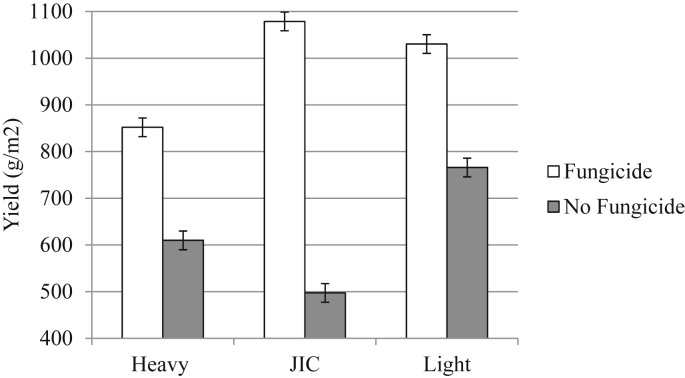
Mean plot yields (g/m^2^) for winter barley variety monocultures and two three-way varietal mixtures in fungicide treated or untreated plots in a field trial at three different sites. N = 144 (N = 24 per monoculture and mixture). Error bars show 95% confidence interval of means.

The JIC site had significantly more brown rust (mean of 17.9% green leaf area covered in symptoms) than the light land (9.3%) and heavy land (15.0%) sites (Fig. 5; Table A4, F_2,15_ = 19.37, *P* < 0.001) and consequently suffered the most from a lack of fungicide application. Non-fungicide trials, had yields less than 50% of those treated with fungicides at this site (Fig. 6).

### 3.3 Lodging

Winsome was very prone to lodging especially on the light sandy soil of JIC (Table A5). Severe lodging of Winsome in monoculture resulted in a very high relative yield of mixtures in fungicide treated plots at JIC most likely as a result of increased plant growth in the absence of disease (Fig. 3). There were no significant differences in plant height at the end of the growing season, but differences between sites were observed at GS 30–32 (Heavy 19 cm; JIC 55 cm; Light 44 cm, complete data not shown). Winsome did not lodge on the heavy land site due to lower plant height in the early part of the season and because fewer pigeons than at JIC flattened the crop to feed on grain close to the ground (personal observation). Mixtures were more resistant to lodging, probably as a result of facilitation. Varieties were not tested in every possible combination so the exact contribution of each variety to lodging in mixtures cannot be calculated. Since Element has strong straw, however, it may have supported neighbouring Winsome plants, suppressing lodging and thus stabilising yield in mixtures (Table A5).

## 4 Discussion

This study investigated how ecological processes act to buffer against environmental stresses and stabilise yield in barley variety mixtures. Such stabilisation occurs in communities of wild plants (Tilman et al., 1996, Hector et al., 1999, Hughes and Stachowicz, 2011) and is expected also to operate in agricultural systems. The stresses included disease, as was predicted, but also unexpected abiotic stresses. Mixture yields were as high as the best performing monocultures, indicating no yield penalty of growing mixtures. Varietal mixtures enhanced spatial yield stability compared to the mean of the component monocultures, supporting the hypothesis that biodiversity increases ecological stability i.e. the ability of an ecological system to maintain or quickly regain productivity despite diverse environmental stresses (Tilman, 1996, Yachi and Loreau, 1999).

In terms of ecological processes, yield stability was largely achieved through compensation and facilitation. In mixtures, the most competitive variety, the hybrid six-row variety Element, which achieved the greatest yields in mixture at a lower seeding rate, compensated for yield losses associated with less competitive varieties such as Cassata. Element was the only variety to display no signs of lodging in monoculture and therefore likely to have contributed to reduced lodging and increased seed production of neighbouring plants in mixtures by facilitation. Previous work with the model plant *Arabidopsis thaliana* demonstrated that genotypes with the highest yield potential are often the most competitive, allowing them to over-yield in mixture through a reduction in the intensity of competition compared to a monoculture of the high-yielding genotype (Creissen et al., 2013, Creissen et al., 2015). In this study, the six-row cultivar, which had the highest yield potential, was indeed responsible for compensation observed within the mixtures.

Ecological processes that contribute towards increased yield stability in mixtures can be identified under laboratory or glasshouse conditions (Creissen et al., 2013), yet experimentation under field conditions may be specific to the variety and particular combination of stresses present. The ecological processes that stabilise yield may differ between sites, but genetic diversity within crops offers the potential for diverse stabilising processes to mitigate the effects of both foreseen and unforeseen stresses. Most major crops (wheat, maize, rice etc.) are grown in environments in which multiple stresses are present that prevent them from achieving their yield potential. Abiotic stresses alone can reduce average yields of most major crop species by more than 50% (Bray et al., 2000), yet plants must cope with stresses such as cold, drought and salinity whilst simultaneously defending themselves from diverse pests and pathogens (Hammond-Kosack and Jones, 2000). Examining stress tolerance by exposing the plant to individual stresses may lead to inaccurate predications, even if care is taken to relate experimental conditions to natural or field conditions (Mittler and Blumwald, 2010). Interactions between biotic and abiotic stresses experienced by plants grown under field conditions can result in varieties responding unpredictably (Mittler, 2006, Atkinson and Urwin, 2012). Abiotic stresses can have positive or negative effects on disease susceptibility in ways difficult to replicate under laboratory or glasshouse conditions. Indeed this study showed high levels of variation in disease severity between geographically similar sites. Studies investigating the effects of multiple stresses on plant productivity and stability under field conditions are valuable for variety mixture and other plant breeding trials as they more accurately represent the unpredictable environmental conditions experienced by crop plants in agricultural systems. Understanding how variation in environments and genotypes within the normal range of modern agriculture affects ecological processes will facilitate the design of trials on the performance and stability of variety mixtures in relation to the full range of important traits.

The majority of empirical studies on variety mixtures have focussed on disease control (Finckh et al., 2000, Zhu et al., 2000, Mundt, 2002), reporting trends in yield and disease severity for the population (Mundt, 2002, Phillips et al., 2005, Newton and Guy, 2009), yet varietal mixtures also offer protection against unexpected stresses related to unpredictable environmental conditions. Few studies have focussed on the plant–plant interactions and ecological processes taking place within mixtures, although there have been studies of crop competition (Allard and Adams, 1969, Finckh and Mundt, 1992) and facilitation (Revilla-Molina et al., 2009). Despite the prediction from the AHDB Recommended List (http://cereals.ahdb.org.uk/varieties/ahdb-recommended-lists/rl-archive-2011-12.aspx) that Element would be the most susceptible to brown rust infection, Winsome was the most susceptible in the trials reported here. While levels of disease in Winsome monocultures were high, brown rust infection was reduced on Winsome in mixtures. This reduction in disease severity may act through a combination of increased distance between the susceptible hosts, and barriers of less susceptible/resistant plants preventing pathogen spread (Chin and Wolfe, 1984, Zhu et al., 2000).

Yield stability was also achieved by unexpected processes in response to unpredicted stresses such as the combination of lodging and herbivory by pigeons. Lodging poses a significant threat to arable production in many areas and can reduce crop yields by up to 60% (Rajkumara, 2008). It continues to be a significant problem in barley cultivation in the UK, especially in winter cultivars. In this study Winsome proved to be highly prone to lodging (especially at JIC) due to a combination of weak straw, irrigation (only at JIC site), early ripening (especially on fungicide-untreated plots), and large numbers of pigeons that further flattened the plants to feed on the grain lying on the ground. The more lodging-resistant varieties, particularly Element, reduced lodging of the entire plant population through facilitation. This variety is recommended by the Agriculture and Horticulture Development Board for use in the north of the UK, where crops are more likely to experience environmental stresses such as rain and high winds. Such stresses increase lodging so a variety with stronger straw, providing increased lodging resistance, is generally favoured. Barley plants in this study experienced conditions more typical of a northern environment, with heavy wind and rain in the weeks prior to harvest that were unusual for East Anglia. The beneficial effect of mixtures on reducing lodging has been observed previously in several crops, including winter barley (Stutzel and Aufhammer, 1989) and rice (Revilla-Molina et al., 2009). As our study was conducted in a single growing season its findings cannot be extrapolated to produce broad conclusions on the ability of these particular mixtures to stabilise productivity over time. Instead our study highlights the fact that the benefits of increased within-crop diversity are not always predictable. Unforeseen environmental stresses due to climate observed in our study highlight the importance of careful mixture selection and experimentation under diverse field conditions for accurate assessment of mixture performance.

Diverse crops offer more capacity for adaptation to both known and unknown stresses, ensuring yield stability over monocultures whilst simultaneously being significantly more practical than alternative methods of increasing within-crop diversity e.g. using composite cross populations (CCP). CCP contain significantly more genetic diversity than a varietal mixtures, but they require breeding efforts and the crops are much less easily manipulated by the farmer. In physical mixtures of varieties each component can be easily substituted on an annual basis. Such modifications to the composition and complexity of the mixture can be made in order to meet specific targets (higher yields, increased grain quality etc.). Varietal mixtures offer advantages over monocultures i.e. increased resilience, yield stability etc. whilst being able to benefit from the advances in plant breeding for monoculture production.

Despite the many advantages of growing barley varietal mixtures, such as increased disease resistance, increased tolerance to abiotic stresses, increased yield and yield stability, adoption of this practice remains restricted (Finckh et al., 2000, Mundt, 2002, Newton et al., 2008a, Newton and Guy, 2009). Mixtures have historically been unacceptable to maltsters and millers on the grounds of grain heterogeneity, grain verification, processing requirements and customer preference. However, grain consistency and grain quality has been shown to be equal to and even better than the sum of the mixture components (Newton et al., 2008b). Approximately 50% of the barley produced in the UK is used for animal feed for which grain consistency is less of a concern than it is for brewers (Brown, 1995, Newton et al., 2011). A major problem of growing mixtures is the uncertainty about the agronomy in which the requirements of multiple varieties must be considered. Variation in heading date between varieties in varietal mixtures may also create problems at harvest. Despite these issues the benefits of growing mixtures include reduced cost of chemical inputs, in turn reducing the cost of crop production. Future cropping systems will need to be less reliant on chemical input, less expensive to manage and show greater ability to cope with the changing environment if future food security is to be achieved (FAO, WFP, IFAD, 2012, Hillocks, 2012). Varietal mixtures designed to exploit beneficial ecological processes such as compensation and facilitation should be able to perform in a wider range of environments and will thus enable farmers to achieve high and stable yields by buffering against diverse and sometimes unpredictable stresses.

## Figures and Tables

**Table A1:** The morphological grain characters used by NIAB to identify the amount of grain contributed by each winter barley variety to the mixture plot yield.

Character	Winsome	Cassata	Saffron	Element
Rachilla hair type	Short	Long	Long	Long
Ventral furrow hair	Absent	Absent	Present	Present
Aleurone colour	White	White	Blue	Blue (weak)
Pigment	Strong	Absent/weak	Absent	Medium
Spicules ILN	Absent/weak	Strong	Medium	Very strong

**Table A2:** The effect of winter barley varietal diversity on plot yield (per 6 m^2^) in a field trial conducted over three different sites. A linear mixed model was used to analyse each factor and all interactions between them. Fixed effects included trial site, fungicide treatment (treated vs. untreated) and Gcomp (Genotypic composition, 6 levels including each monoculture and both mixtures). Non-significant terms were eliminated from the model. *F* and *P* values refer to ANOVA tests of each factor separately and the interactions between them. N = 144 (N = 24 per monoculture/mixture).

Fixed term	F	n.d.f.	d.d.f.	P
Site	15.34	2	94.9	<0.001
Fungicide	156.36	1	50.5	<0.001
Gcomp	2.23	5	53.5	0.141
Site.Fungicide	18.43	2	95.5	<0.001

**Table A3:** The effect of four winter barley varietal diversity on a) mean mass per ear and b) mean mass per grain, in a field trial conducted over three different sites. A linear mixed model was used to analyse each factor and all interactions between them. Fixed effects included trial site, fungicide treatment (treated vs. untreated) and cultivation (monoculture/mixture). Non-significant terms were eliminated from the model. *F* and *P* values refer to ANOVA tests of each factor separately and the interactions between them. N = 240 per variety.

Fixed term	F	n.d.f	d.d.f	P
a)				
Site	0.42	2	3587	0.516
Fungicide	6.86	1	3582	0.009
Cultivation	30.79	1	3583	<0.001
Variety	90.85	3	3582	<0.001
Cultivation.Variety	3.6	3	3581	0.013
b)				
Site	0.42	2	3587	0.5
Fungicide	6.88	1	3562	0.0009
Cultivation	30.87	1	3563	<0.001
Variety	91.07	3	3560	<0.001

**Table A4:** The effect of winter barley varietal diversity on brown rust disease severity (measured as % green leaf area covered in disease on the flag and second leaf) in a field experiment conducted over three different sites. A linear mixed model was used to analyse each factor and all interactions between them. Fixed effects included trial site, cultivation and variety (mixture/monoculture). Non-significant terms were eliminated from the model. *F* and *P* values refer to ANOVA tests of each factor separately and the interactions between them. N = 1680.

Fixed term	F	n.d.f.	d.d.f	P
Site	19.37	2	144	<0.001
Variety	13.39	3	144	<0.001
Cultivation	0.2	1	144	0.652
Site.Variety	2.37	6	144	0.033
Variety.Cultivation	4.31	3	144	0.006

**Table A5:** Percentage and standard deviations of plots that were fully lodged (75–90° from upright) on 25/06/12 in a field trial of mixtures and monocultures of winter barley. Lodging was estimated by eye to the nearest one-eighth of a plot. Plots not represented showed no signs of lodging. N = 144.

Site	Cultivation	Plot	Fungicide	% Lodged	St. Dev.
JIC	Mono	Winsome	No fungicide	100	0
JIC	Mono	Winsome	Fungicide	100	0
JIC	Mono	Cassata	No fungicide	12.5	14.4
JIC	Mono	Saffron	No fungicide	9.4	12
JIC	Mix	A	No fungicide	50	20.4
JIC	Mix	A	Fungicide	25	20.4
JIC	Mix	B	No fungicide	25	20.4
JIC	Mix	B	Fungicide	25	20.4
Light	Mono	Winsome	No fungicide	25	0
Light	Mono	Winsome	Fungicide	12.5	0

**Table 1:** Information from HGCA Recommended List 2011/2012. Ratings for the winter barley varieties used in this study: 1 = poor resistance to disease or stress, 9 = high resistance. Mixture A includes varieties Element, Winsome and Saffron. Mixture B includes varieties Element, Winsome and Cassata.

Variety	Rhyncho-sporium	Brown rust	Net blotch	Mildew	Lodging	Straw height (cm)	Yield with fungicide (t/ha)	Yield no fungicide (t/ha)
Element	7	4	7	6	6	103	9.3	7.7
Winsome	8	6	8	7	6	93	8.6	7.0
Saffron	4	7	4	3	8	87	9.0	7.1
Cassata	8	7	8	4	8	87	8.5	7.0

## References

[bib1] Allard R.W., Adams J. (1969). Population studies in predominantly self-pollinating species. 13. Intergenotypic competition and population structure in barley and wheat. Am. Nat..

[bib2] Annicchiarico P. (2002). Genotype x environment interaction: challenges and opportunities for plant breeding and cultivar recommendations (English). FAO Plant Production and Protection Paper (FAO).

[bib3] Atkinson N.J., Urwin P.E. (2012). The interaction of plant biotic and abiotic stresses: from genes to the field. J. Exp. Bot..

[bib4] Becker H.C., Léon J. (1988). Stability analysis in plant breeding. Plant Breed..

[bib5] Bray E.A., Bailey-Serres J., Weretilnyk E., Gruissem W., Buchannan B., Jones R. (2000). Responses to abiotic stresses. Biochemistry and Molecular Biology of Plants.

[bib6] Brown J.K.M. (1995). 5 Pathogens' responses to the management of disease resistance genes. Adv. Plant Pathol..

[bib7] Callaway R.M. (1995). Positive interactions among plants. Bot. Rev..

[bib8] Chin K.M., Wolfe M.S. (1984). The spread of *Erysiphe-graminis* f.sp. *hordei* in mixtures of barley varieties. Plant Pathol..

[bib9] Cowger C., Weisz R. (2008). Winter wheat blends (mixtures) produce a yield advantage in north Carolina. Agron. J..

[bib10] Creissen H.E., Jorgensen T.H., Brown J.K.M. (2013). Stabilisation of yield in plant genotype mixtures through compensation rather than complementation. Ann. Bot..

[bib11] Creissen H.E., Jorgensen T.H., Brown J.K.M. (2015). Impact of disease on diversity and productivity of plant populations. Funct. Ecol..

[bib12] de Wit C.T. (1960). On competition. Verslag Landbouwk Onderz..

[bib13] Eberhart S.A., Russell W.A. (1966). Stability parameters for comparing yield. Crop Sci..

[bib14] FAO, WFP, IFAD (2012). The State of Food Insecurity in the World 2012. Economic Growth Is Necessary but Not Sufficient to Accelerate Reduction of Hunger.

[bib15] Finckh M.R., Gacek E.S., Goyeau H. (2000). Cereal variety and species mixtures in practice, with emphasis on disease resistance. Agronomie.

[bib16] Finckh M.R., Mundt C.C. (1992). Plant competition and disease in genetically diverse wheat populations. Oecologia.

[bib17] Finckh M.R., Wolfe M.S., Cooke B.M., Jones G.D., Kaye B. (1998). Diversification strategies. The Epidemiology of Plant Diseases.

[bib18] Hammond-Kosack K.E., Jones J.D.G., Buchannan B., Gruissem W., Jones R. (2000). Response to plant pathogens. Biochemistry and Molecular Biology of Plants.

[bib19] Hector A., Schmid B., Beierkuhnlein C. (1999). Plant diversity and productivity experiments in European grasslands. Science.

[bib20] Hillocks R.J. (2012). Farming with fewer pesticides: EU pesticide review and resulting challenges for UK agriculture. Crop Prot..

[bib21] Hughes A.R., Stachowicz J.J. (2011). Seagrass genotypic diversity increases disturbance response via complementarity and dominance. J. Ecol..

[bib22] James W.C., Jenkins J.E.E., Lemmett J.L. (1968). The relationship between leaf blotch caused by Rhynchosporium secalis and losses in grain yield of spring barley. Ann. Appl. Biol..

[bib23] James W.C. (1971). An illustrated series of assessment keys for plant diseases their preparation and usage. Can. Plant Dis. Surv..

[bib24] Lannou C., Mundt C.C. (1996). Evolution of a pathogen population in host mixtures: simple race-complex race competition. Plant Pathol..

[bib25] Lopez C.G., Mundt C.C. (2000). Using mixing ability analysis from two-way cultivar mixtures to predict the performance of cultivars in complex mixtures. Field Crops Res..

[bib26] Loreau M. (2000). Biodiversity and ecosystem functioning: recent theoretical advances. Oikos.

[bib27] Mille B., Fraj M.B., Monod H., de Vallavieille-Pope C. (2006). Assessing four-way mixtures of winter wheat cultivars from the performances of their two-way and individual components. Eur. J. Plant Pathol..

[bib28] Mittler R. (2006). Abiotic stress, the field environment and stress combination. Trends Plant Sci..

[bib29] Mittler R., Blumwald E. (2010). Genetic engineering for modern agriculture: challenges and perspectives. Ann. Rev. Plant Biol..

[bib30] Mulder C.P.H., Uliassi D.D., Doak D.F. (2001). Physical stress and diversity-productivity relationships: the role of positive interactions. Proc. Natl. Acad. Sci..

[bib31] Mundt C.C. (2002). Use of multiline cultivars and cultivar mixtures for disease management. Annu. Rev. Phytopathol..

[bib32] Newton A.C., Begg G.S., Swanston J.S. (2008). Deployment of diversity for enhanced crop function. Ann. Appl. Biol..

[bib33] Newton A.C., Flavell A.J., George T.S. (2011). Crops that feed the world 4. Barley: a resilient crop? Strengths and weaknesses in the context of food security. Food Secur..

[bib34] Newton A.C., Guy D.C. (2009). The effects of uneven, patchy cultiver mixtures on disease control and yield in winter barley. Field Crops Res..

[bib35] Newton A.C., Hackett C.A., Swanston J.S. (2008). Analysing the contribution of component cultivars and cultivar combinations to malting quality, yield and disease in complex mixtures. J. Sci. Food Agric..

[bib36] Østergård H., Kristensen K., Jensen J.W. (2005). Stability of Variety Mixtures of Spring Barley. in: Proceedings of the COST SUSVAR/ECO-PB. Workshop on Organic Plant Breeding Strategies and the Use of Molecular Markers Driebergen.

[bib37] Peterson R.F., Campbell A.B., Hannah A.E. (1948). A diagrammatic scale for estimating rust intensity on leaves and stems of cereals. Can. J. Res. Sect. C-Botanical Sci..

[bib38] Phillips S.L., Shaw M.W., Wolfe M.S. (2005). The effect of potato variety mixtures on epidemics of late blight in relation to plot size and level of resistance. Ann. Appl. Biol..

[bib39] Rajkumara S. (2008). Lodging in cereals - a review. Agric. Rev..

[bib40] Revilla-Molina I.M., Bastiaans L., Van Keulen H. (2009). Does resource complementarity or prevention of lodging contribute to the increased productivity of rice varietal mixtures in Yunnan, China. Field Crops Res..

[bib41] Sapoukhina N., Paillard S., Dedryver F., de Vallavieille-Pope C. (2013). Quantitative plant resistance in cultivar mixtures: wheat yellow rust as a modeling case study. New Phytol..

[bib42] Smithson J.B., Lenne J.M. (1996). Varietal mixtures: a viable strategy for sustainable productivity in subsistence agriculture. Ann. Appl. Biol..

[bib43] Stutzel H., Aufhammer W. (1989). Effects of winter barley cultivars on lodging. J. Agric. Sci..

[bib44] Tilman D. (2004). Niche tradeoffs, neutrality, and community structure: a stochastic theory of resource competition, invasion, and community assembly. Proc. Natl. Acad. Sci..

[bib45] Tilman D. (1996). Biodiversity: population versus ecosystem stability. Ecology.

[bib46] Tilman D., Wedin D., Knops J. (1996). Productivity and sustainability influenced by biodiversity in grassland ecosystems. Nature.

[bib47] VSN International (2011).

[bib48] Wolfe M.S., Barrett J.A. (1980). Can we lead the pathogen astray. Plant Dis..

[bib49] Wolfe M.S. (1985). The current status and prospects of multiline cultivars and variety mixtures for disease resistance. Annu. Rev. Phytopathol..

[bib50] Wricke G. (1962). Über eine methode zur erfassung der ökologischen Streubreite infeldversuchen. Z. Pflanzenzüchtg.

[bib51] Yachi S., Loreau M. (1999). Biodiversity and ecosystem productivity in a fluctuating environment: the insurance hypothesis. Proc. Natl. Acad. Sci..

[bib52] Zadoks J.C., Chang T.T., Konzak C.F. (1974). A decimal code for the growth stages of cereals. Weed Res..

[bib53] Zhu Y.Y., Chen H.R., Fan J.H. (2000). Genetic diversity and disease control in rice. Nature.

